# Development of an IoT-Based Flood Monitoring System Integrated with GIS for Lowland Agricultural Areas

**DOI:** 10.3390/s25175477

**Published:** 2025-09-03

**Authors:** Sittichai Choosumrong, Kampanart Piyathamrongchai, Rhutairat Hataitara, Urin Soteyome, Nirut Konkong, Rapikorn Chalongsuppunyoo, Venkatesh Raghavan, Tatsuya Nemoto

**Affiliations:** 1 Department of Natural Resources and Environment, Faculty of Agriculture, Natural Resources and Environment, Naresuan University, Phitsanulok 65000, Thailand; sittichaic@nu.ac.th (S.C.); rhutairath@gmail.com (R.H.); 2 Center of Excellence in Nonlinear Analysis and Optimization, Faculty of Science, Naresuan University, Phitsanulok 65000, Thailand; 3 Department of Water Management and Maintenance, Regional Irrigation Office 3—Royal Irrigation Department, Phitsanulok 65000, Thailand; urinsoteyome@gmail.com (U.S.); nirut.k@ku.th (N.K.); 4 Faculty of Education, Nakhon Sawan Rajabhat University, Nakhon Sawan 60000, Thailand; milk20715@hotmail.com; 5 Graduate School of Science, Osaka Metropolitan University, Osaka 558-8585, Japan; raghavan@omu.ac.jp

**Keywords:** flood monitoring, spatial data analysis, low-cost sensor, Internet of Things

## Abstract

Disaster risk reduction requires efficient flood control in lowland and flood-prone areas, especially in agricultural areas like the Bang Rakam model area in Phitsanulok province, Thailand. In order to improve flood prediction and response, this study proposes the creation of a low-cost, real-time water-level monitoring integrated with spatial data analysis using Geographic Information System (GIS) technology. Ten ultrasonic sensor-equipped monitoring stations were installed thoughtfully around sub-catchment areas to provide highly accurate water-level readings. To define inundation zones and create flood depth maps, the sensors gather flood level data from each station, which is then processed using a 1-m Digital Elevation Model (DEM) and Python-based geospatial analysis. In order to create dynamic flood maps that offer information on flood extent, depth, and water volume within each sub-catchment, an automated method was created to use real-time water-level data. These results demonstrate the promise of low-cost IoT-based flood monitoring devices as an affordable and scalable remedy for communities that are at risk. This method improves knowledge of flood dynamics in the Bang Rakam model area by combining sensor technology and spatial data analysis. It also acts as a standard for flood management tactics in other lowland areas. The study emphasizes how crucial real-time data-driven flood monitoring is to enhancing early-warning systems, disaster preparedness, and water resource management.

## 1. Introduction

The Royal Irrigation Department (RID), through the Regional Irrigation Office 3 in Phitsanulok Province, has implemented the Bang Rakam model project, focusing on strategic water management in lowland areas. Each year, the project adjusts the planting schedule for rain-fed rice farming to begin in April and complete harvesting by July, allowing the area to function as a temporary flood retention zone during the rainy season. The Bang Rakam model area is capable of storing approximately 400–500 million cubic meters of water; however, this capacity is estimated based on the experience of irrigation officials and local residents as precise flood volume measurements in such an expansive area remain challenging [1]. Current flood monitoring tools in the region still exhibit discrepancies in measuring flood volume, leading to delays in flood warnings and inefficiencies in emergency response due to the lack of real-time flood data [2]. Recent studies emphasize the importance of accurate flood volume assessment and real-time data collection in flood-prone regions [3,4,5,6].

A field survey conducted by the Irrigation Region 3 Office during the 2020 rainy season revealed that local residents were significantly affected by the lack of reliable flood data. This deficiency led to poor agricultural and aquaculture planning, including delays in seedling preparation and fishing activities, as farmers were unaware of the actual water situation [7]. Furthermore, the lack of public participation in water management was in part due to the inaccessibility of flood data and monitoring technologies [8]. Studies highlight that limited accessibility to real-time flood data hinders effective decision-making and planning, particularly in rural agricultural communities.

In recent years, the use of Synthetic Aperture Radar (SAR) data for flood detection and mapping has grown significantly, driven by the increased availability of recent missions offering improved temporal resolution [9,10,11,12]. For example, Notti et al. [13] presents a semi-automatic, low-cost, and user-friendly method that integrates free multispectral data (MODIS, Proba-V, Landsat, Sentinel-2) and SAR data (Sentinel-1) with open-source tools, validated against official flood maps. This approach is particularly advantageous in areas with limited data or resources due to its integration of diverse satellite sources, cost-efficiency, and validation capabilities. However, limitations still exist, including the coarse resolution of sensors like MODIS and Proba-V, cloud obstruction in optical data, and dependency on satellite revisit intervals. These factors can hinder timely and accurate flood detection in rapidly changing situations, making it challenging to provide the granular, real-time insights needed for localized agricultural planning in areas like Bang Rakam. In contrast, the proposed system employs affordable ultrasonic water-level sensors connected to battery-powered microcontrollers and 3G pocket WiFi devices—an effective solution for rural Thai areas lacking LoRa infrastructure. While this system does not offer the broad spatial coverage of satellite remote sensing, it enables continuous, near real-time local data collection with higher temporal resolution. This continuous, hyper-local data is particularly critical for the Bang Rakam context, supporting early warnings and accurate flood volume estimation essential for detailed agricultural and aquaculture planning that satellite data alone cannot provide. Moreover, by integrating sensor data with high-resolution (1-m) DEMs and geospatial analysis using Python (version 3.9.7), the system generates detailed and timely flood depth maps, effectively bridging the gap between broad satellite observations and precise ground-based measurements. To address this issue, the Regional Irrigation Office 3 developed a semi-automatic water-level assessment tool based on closed-circuit television (CCTV) images. This system, referred to as Semi-GIC (Graphic Information Camera), relies on manual evaluation by personnel to determine water levels from CCTV footage [14,15,16]. However, this semi-automated process is labor-intensive and dependent on staff availability, particularly during non-office hours, which limits the timeliness and efficiency of data collection. Some studies [17,18,19] show that IoT-based systems that use automation and artificial intelligence (AI) can solve these problems by reducing human labor and enabling constant, real-time flood monitoring [20,21].

The review of various techniques and methods from previous studies has revealed the limitations and challenges associated with each approach; therefore, this research clearly focuses on the development of a real-time flood monitoring and mapping system for lowland agricultural areas. The core objective is not the design of novel sensor hardware but rather the integration of existing low-cost IoT sensors with GIS-based spatial analysis to enhance flood prediction and response [22]. By combining water-level sensors with real-time data transmission and raster-based flood simulation, the system aims to generate accurate flood extent maps, support early-warning mechanisms, and improve decision-making in flood-prone regions [21,23]. This approach addresses current limitations in traditional flood monitoring systems, particularly in under-resourced settings [24].

## 2. Materials and Methods

**Figure 1:**
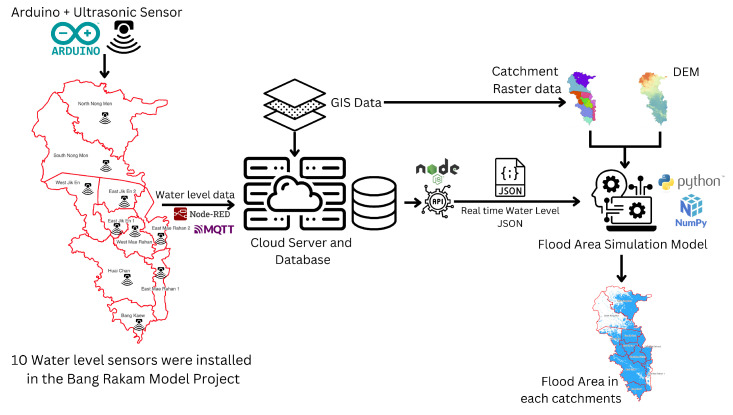
This figure shows the framework of this study.

To effectively monitor flood dynamics in the Bang Rakam model area, this study integrates low-cost IoT-based water-level sensors with a flood simulation model [25]. The approach involves deploying sensors across multiple stations to collect high-frequency water-level data, which is then processed and analyzed to generate spatial flood extent and depth maps [16,26]. By combining sensor-based measurements with geospatial analysis, the system provides a cost-effective and automated alternative to traditional flood monitoring method [27,28,29]. The following sections detail the sensor deployment strategy and the flood model development process, describing the hardware components, data transmission methods, and analytical techniques used in this study. Figure 1 demonstrates the concept of this framework; 10 water-level stations, coupled with Arduino and ultrasonic technology, have been installed in the Bang Rakam model area. These sensors collect water-level data and send it to a cloud server using Message Queuing Telemetry Transport (MQTT) protocol, which uses Node-RED for data management and API interaction [30]. The cloud architecture saves and processes water-level data at predetermined time intervals, sending JSON outputs to modeling applications. Python-based flood simulation models, powered by libraries like NumPy, combine GIS data, such as catchment raster data and DEM, with water-level data. In this study, the DEM used for topographic analysis and floodplain modeling was obtained from the Regional Irrigation Office 3, which provided high-resolution elevation data critical for accurately simulating water flow and inundation patterns. Specifically, the elevation data corresponds to a Digital Terrain Model (DTM), which represents the bare-earth surface without including buildings, vegetation, or other above-ground features, ensuring more precise hydrological modeling than a Digital Surface Model (DSM) would allow. The produced models predict flood-prone areas in all catchments, resulting in flood-prone maps. These catchments were delineated through hydrological analysis performed on the DEM using r.basin in GRASS GIS.

### 2.1. Study Area

The provinces of Sukhothai and Phitsanulok are extremely vulnerable to floods, especially in the Yom River Basin’s lowland regions. Because of the basin’s conical and twisting topography, water drainage is considerably slowed, which prolongs flooding throughout the rainy season. Excess water frequently overflows downstream from heavy rainfall in the upper Yom River Basin, causing significant flooding in Sukhothai province that affects both residential areas and agricultural grounds [1].

**Figure 2:**
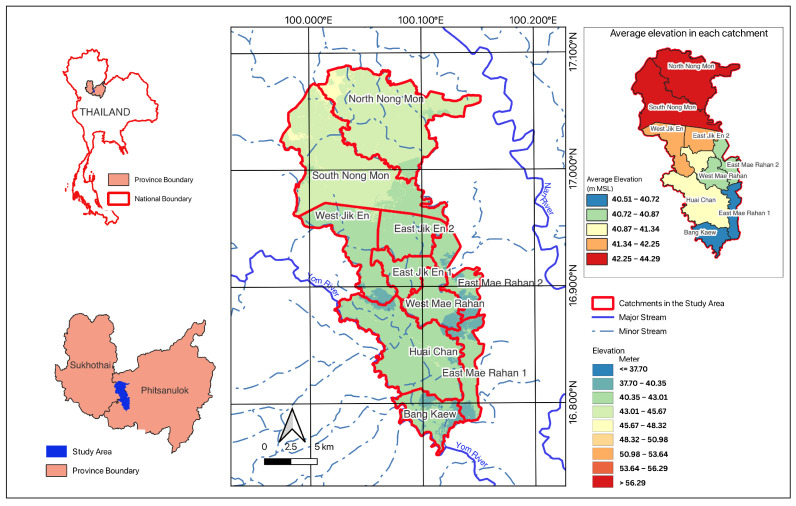
Study area.

The Bang Rakam model area, as shown in Figure 2, is located in lower Phitsanulok province and serves as a designated flood retention zone. This region is primarily a floodplain, characterized by vast expanses of rice paddies and low-lying farmland that are prone to seasonal flooding. The area’s natural topography allows it to temporarily store floodwaters before gradual drainage into the Nan River system, reducing the severity of flooding in downstream areas. Encompassing approximately 360 square kilometers, this strategic area is situated between two major rivers: the Yom River and the Nan River. Within the study area, a dense network of canals crisscrosses the landscape. The average elevation of the Bang Rakam model area is approximately 41 m above mean sea level (m MSL), with elevations ranging from a minimum of 38 m MSL to a maximum of approximately 59 m MSL. The area receives an average annual rainfall of approximately 1297.513 mm, with the highest precipitation typically occurring from September to October. The study area features a network of irrigation canals, reservoirs, and floodplains, which play a crucial role in water retention and distribution. These water bodies influence the spatial and temporal variations in flood depth and duration, making it essential to understand their hydrological interactions for effective flood assessment and management.

### 2.2. Hardware Design and Implementation

The goal of developing and implementing a water-level monitoring system is to provide a reliable and effective platform for precisely tracking water levels. Modern technologies like low-cost sensors for real-time data collecting, automation to lessen the need for manual work, and sophisticated geospatial analysis tools for efficient data processing and visualization are all integrated into this system [27,31]. The created system is to improve flood monitoring [32], increase water management capabilities, and offer timely information for decision-making in lowland areas that are vulnerable by resolving current restrictions.

**Figure 3:**
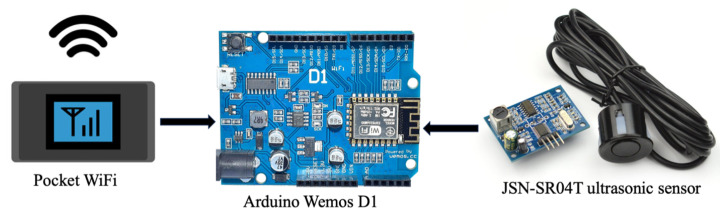
Show the system architecture of ultrasonic distance measurement and communication.

The creation of the real-time water-level monitoring system, which makes use of the JSN-SR04T Ultrasonic Module, is shown in Figure 3. This module is appropriate for outdoor applications because of its splash and moisture resistance construction. The sensor can identify objects and determine their distance from the sensor, which is between 20 cm and 4 m. With a working current of less than 8 mA, a probe frequency of 40 kHz, a resolution of 1 mm, a distance accuracy of ±1 cm, and a DC voltage of 3.0 to 5.5 V, it functions. Because of these features, the module is very dependable for precise and effective water-level readings in real-time monitoring systems.

As illustrated in Figure 3, the sensor is combined with an Arduino Wemos D1 ESP8266 board, a Wi-Fi-capable board built around the ESP8266 chip. The Wemos D1 runs at 3.3 V and has the same design as the Arduino UNO. The board is controlled by the 32-bit CPU ESP8266 chip, which also has more flash memory than the Arduino UNO. With its eleven digital I/O pins and one analog input pin, it can be used in a variety of ways. A Micro-B USB cable can be used to connect the board. It is a great platform for IoT systems because of its Wi-Fi capability and support for MQTT communication, which allows it to effectively control outputs, read inputs, and manage interruptions [33,34]. This configuration allows for smooth data transfer between IoT devices and the central server in real-time via 3G signals supplied by Pocket Wi-Fi. Each station’s equipment consumes approximately 15 watts per hour. To ensure adequate power supply during nighttime when solar charging is unavailable, a 12 voltage (V) 20 ampere-hour (Ah) sealed lead-acid (SLA) battery was employed. The battery has a total capacity of 240 Watt-hour (Wh) and can power the system for roughly 15 h, which covers the typical 11–12 h period without sunlight, as described in Equation (1). To ensure reliable daily recharging, a 100 watt (W) solar panel with a maximum power voltage (Vmp) of 18 V was used, along with a 30 ampere (A) solar charge controller. In practical conditions, a full-power output from the panel can be achieved only during peak sunlight hours (e.g., 11:00 a.m.–2:00 p.m.), and actual usable power is around 70–80%, or approximately 75 W, due to efficiency loss. Therefore, based on Equation (2), the battery can be fully charged in approximately 3–4 h, which is sufficient for operational requirements.

(1) Power (W)=Voltage (V)×Current (A)

(2) $$\mathrm{Charging}\mathrm{Time}\;\left(\mathrm{hours}\right)=\frac{\mathrm{Battery}\mathrm{Capacity}\;(\mathrm{Wh})}{\mathrm{Actual}\mathrm{Power}\mathrm{Output}\mathrm{from}\mathrm{Solar}\mathrm{Panel}\;(W)}$$

**Figure 4:**
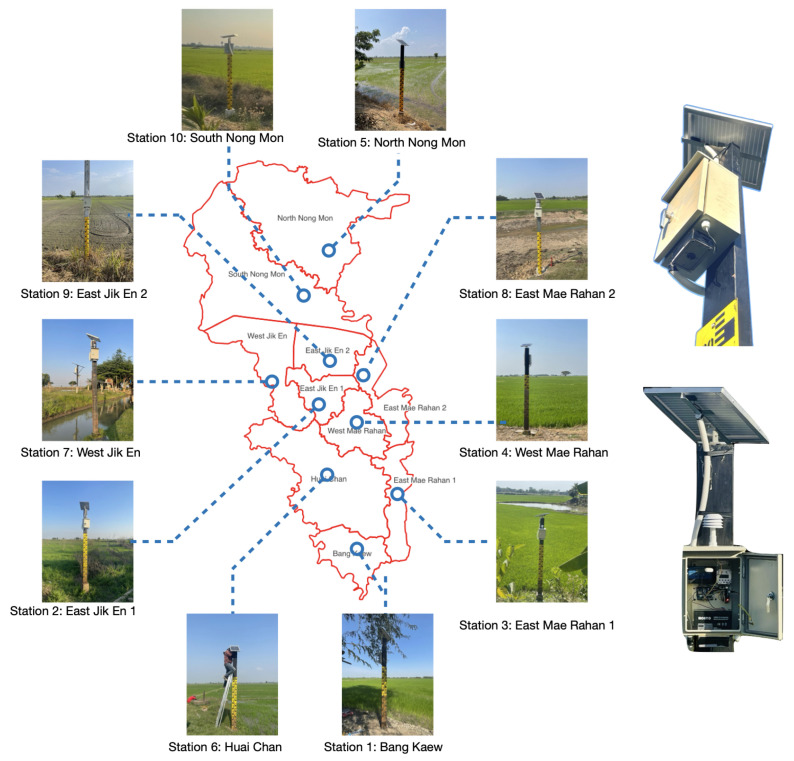
Deployment of water-level monitoring stations in the Bang Rakam model area.

As seen in Figure 4, all ten stations were placed in the Bang Rakam model area, with sensor locations determined based on field surveys conducted by the Regional Irrigation Office 3. The site selection criteria began with identifying the lowest elevation point within each catchment area to ensure early detection of floodwater accumulation. Following this, the feasibility of installation was assessed based on accessibility, signal availability, and the ability to obtain permission from local landowners or stakeholders. Details of the installation locations are provided in Table 1.

For high-frequency water-level monitoring, a sensor unit consisting of a JSN-SR04T ultrasonic sensor is placed 350 cm above the surface. The sensor was manually maintained once a week and included a waterproof glue covering to guard against damage from extended exposure to water. In order to avoid system freezes and guarantee smooth functioning, a timer was also built to reset the sensor on a regular basis. To reduce the possibility of equipment damage, all parts were kept inside a weatherproof enclosure that protected them from elements including sunshine, wind, and rain.

### 2.3. Real-Time Data Transmission and Storing

**Figure 5:**
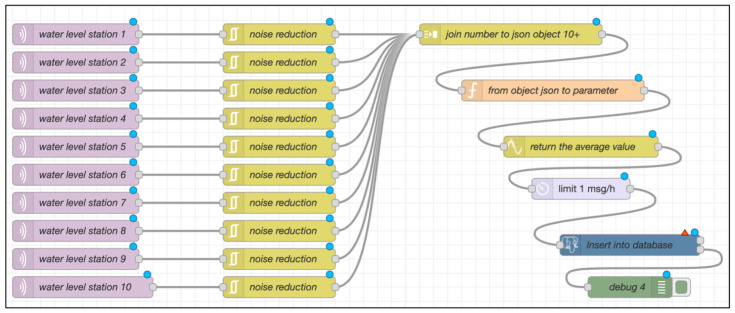
Data processing and transmission workflow using Node-RED.

Since the RID releases water into the area and diverts it out around this time every year, we began gathering data from the beginning of August until the second week of November. A real-time data transmission module that uses a 3G network and an Arduino Wemos D1 ESP8266 board to transmit data over the MQTT protocol to a central server. The sensor devices use the MQTT protocol to transmit data when they are connected to a pocket Wi-Fi network. Figure 5 illustrates how Node-RED, which subscribes to MQTT, processes the data before sending it to the PostgreSQL/PostGIS database. Table 2 describes the structure of the sensor data table used in the database. Each record stores distance measurements associated with a specific station (id_station), along with the corresponding timestamp. The field distance represents the measured distance from the sensor to the water surface.

The system was tested and installed over a 12-month period, as summarized in Table 3. Data transmission is configured to occur once daily during the initial phase when water has not yet entered the area (January–July), primarily to test and verify the system’s functionality of power, Wi-Fi signal, and battery without using any water-level thresholds. Once water begins to flood into the area (August), the transmission frequency is manually adjusted to once every hour to enable near real-time monitoring. This adjustment is made by modifying the limit function within the Node-RED workflow, allowing for flexible control of data transmission rates based on changing flood conditions. Spatial analysis techniques are used to further evaluate the data once it has been placed in the database. An example of the Node-RED workflow is shown in Figure 5, where the parameter limit is set to one message per hour (msg/h), allowing data transfer to happen once every hour. The average value determined over the course of an hour is represented by each transmitted message.

### 2.4. Flood Simulation Model

**Figure 6:**
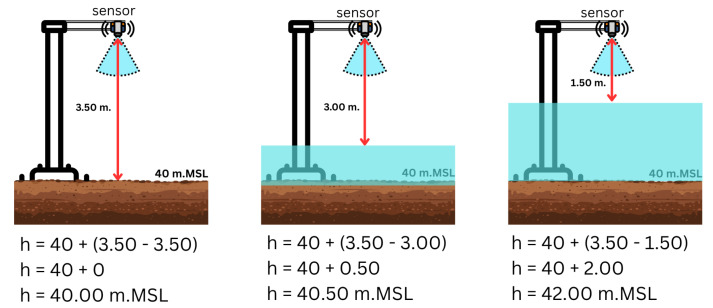
Illustration of the calculation of elevation values from the sensor-measured distance into meters above mean sea level (m.MSL).

A flood simulation model was created utilizing water-level data obtained from ten sensor locations in the Bang Rakam model area. The system incorporates a variety of data sources, including real-time water-level data from sensors, raster-based catchment area information, and DEM data [4,5]. These datasets are run via a simulation algorithm that predicts flood extent and depth in the selected catchment regions. Figure 6 illustrates an example of how water surface elevation (in meters above mean sea level, m.MSL) is calculated using distance measurements from the sensor based on a known ground elevation of 40.00 m.MSL and a fixed sensor height of 3.50 m above the ground; as the water-level rises, the measured distance decreases accordingly, allowing for precise computation of water surface elevation.

**Figure 7:**
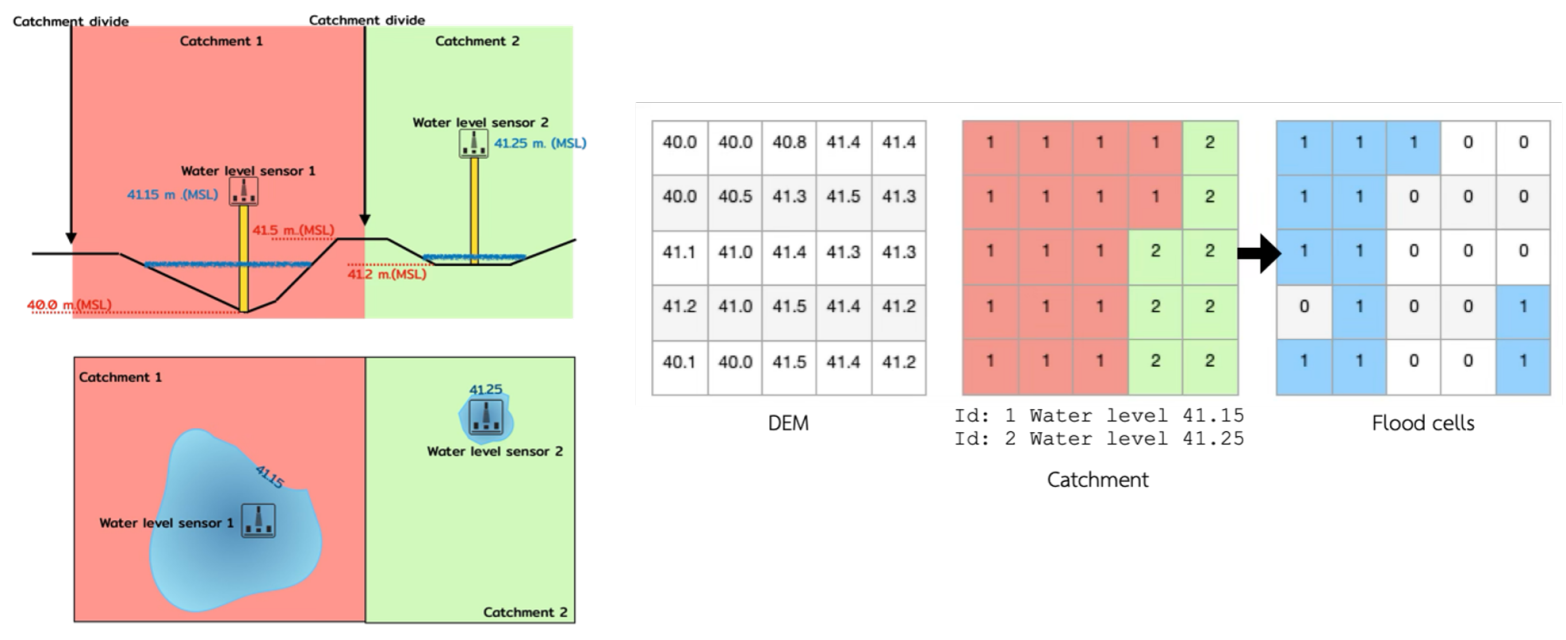
Flood-area analysis model.

Figure 7 explains how to generate DEM and catchment area data in raster format to suit grid observations from 10 monitoring stations. The application examines this information by comparing DEM values for each grid cell to actual water levels. If a DEM cell value is less than or equal to the recorded water level, it is deemed flooded; otherwise, it remains dry. The simulation results are demonstrated by mapping the flooded areas, indicating flood extent using data collected from sensors.

For flood-area simulation, the following steps were created using raster-based processing [3]:

- Data Preparation: Gather raster-based catchment data with corresponding dimensions and DEM data.
- Water-Level Retrieval: Get data from 10 sensors about the water level in JSON format by retrieving it from the database. The simplest method for retrieving data from the server for additional analysis is to use the JSON format [35,36].
- Data processing: The DEM and catchment data are loaded as Numpy arrays using Python programming to do computations.
- Flood Calculation: Depending on the level of each catchment region, designate a cell as flooded (1) if its DEM value is less than the sensor water level and as dry (0) otherwise.
- Generating Results: Save the processed data in Geotiff format for display.

## 3. Results

**Figure 8:**
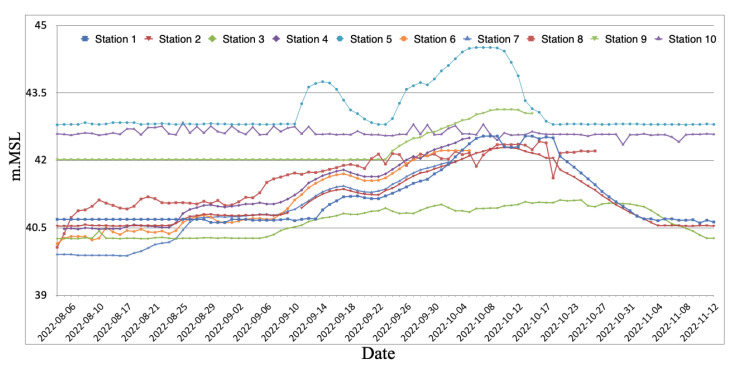
Water-level trends across monitoring stations.

An overview of the daily water-level readings and depth fluctuations obtained at every monitoring station in the Bang Rakam model region is given in Figure 8. With recorded water levels ranging from 40 to 44 m above the mean sea level (m.MSL), the data shows that overall water levels started to rise in the first week of September and then progressively dropped to near-normal levels between early and mid-November. With a maximum recorded water level of around 44.51 m.MSL and a ground height of about 42.81 m.MSL, Station 5 (North Nong Mon) is the highest elevation of the monitoring stations. With a highest recorded water level of 41.96 m.MSL and a ground height of 39.9 m.MSL, Station 7 (East Jik En) is situated at the lowest elevation. The disparities in flood behavior throughout the region are emphasized by these water-level fluctuations.

Because there are ten different catchments in the research area, the graph shows how the initial water levels before flooding vary in m.MSL. Users can monitor the data in real time. Creating an appropriate flood model that takes into consideration the variations in water flow dynamics is made more difficult by this spatial diversity. Furthermore, the graph shows that the timing of water entry and outflow varies among catchments, suggesting that drainage and flooding do not always happen at the same time.

This asynchronous behavior is further influenced by the relatively flat topography of the study area, where elevation differences between basins are minimal. Flooding often begins in the southern catchments—such as West Mae Rahan, East Mae Rahan 1 and 2, Huai Chan, and Bang Kaew—which are located at lower elevations and situated closer to the natural drainage outlets like the Yom River. In these areas, water typically starts to overflow from minor streams when the capacity is exceeded, leading to early inundation. These catchments function as natural accumulation zones where upstream runoff converges, causing them to experience early-stage flooding despite their downstream position. This demonstrates how intricate the water movement is in the Bang Rakam model and emphasizes the necessity of a thorough flood simulation to comprehend and forecast flood behavior in the area.

**Figure 9:**
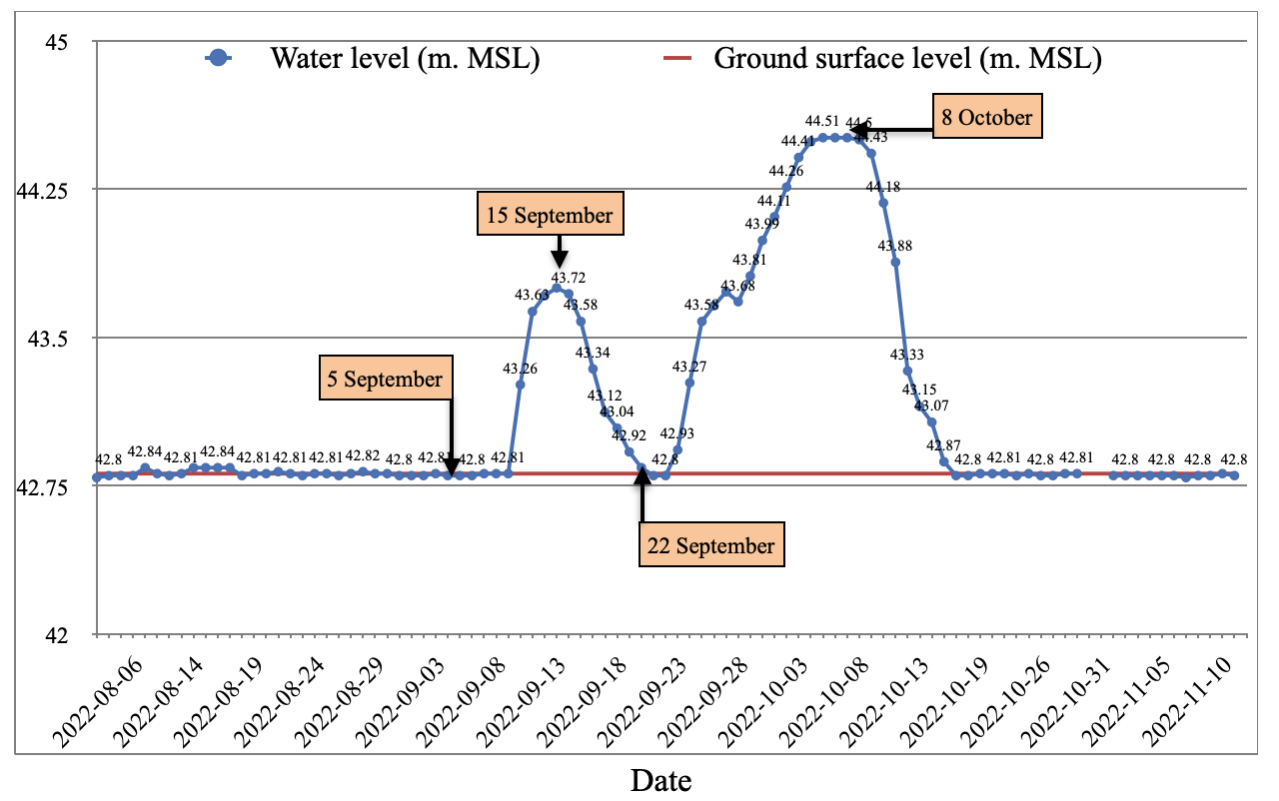
Water-level variation at station 5.

A graph of water-level readings taken at Station 5 is shown in Figure 9, which illustrates how the water level (measured in m.MSL) has changed over time. The red line indicates the station’s ground surface level, and the blue line shows variations in the water level. According to the graph, water levels first stayed constant before beginning to rise in mid-September and peaking at about 44.51 m.MSL in early October before progressively dropping back to normal levels in mid-November. With separate input and outflow times, the observed pattern accurately depicts the seasonal flood dynamics in the study area.

**Figure 10:**
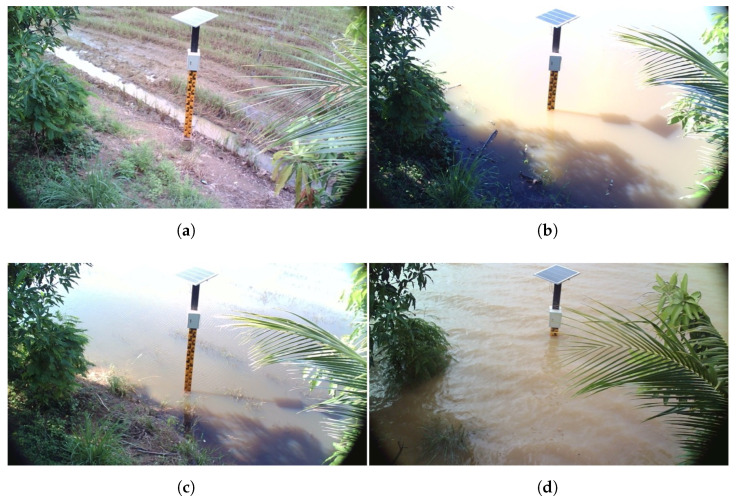
Visual water-level observations at Station 5: (**a**) showing the progression of flooding on 5 September 2022, (**b**) 15 September 2022, (**c**) 22 September 2022, and (**d**) 8 October 2022.

A visual record of the flooding advancement on 5 September, 15 September, 22 September, and 8 October is shown in Figure 10, which is taken from the monitoring camera at Station 5. The sensor is positioned in a rice field in the first photograph (5 September), which depicts a dry environment. Water gradually submerges the staff gauge as the flooding worsens, and on 8 October, the water level is significantly higher, matching the peak levels shown in Figure 9. The automatic water-level monitoring system’s dependability is shown by these pictures, which visibly validate the sensor data. These images together show how the sensor-based monitoring system efficiently records flood dynamics, offering both visual and numerical confirmation—both of which are essential for flood control and decision-making in the Bang Rakam model region.

Following collection, the water-level data is incorporated into the flood model for additional examination. The model utilizes the reported water levels to simulate flood extent, depth variations, and drainage patterns across the study area. The flood model offers a thorough depiction of water movement by integrating temporal and spatial data, enabling a more precise evaluation of flood-prone areas. In the Bang Rakam model area, this data is crucial for early-warning systems, disaster management, and enhancing water management plans.

### 3.1. Simulate Flood Map

**Figure 11:**
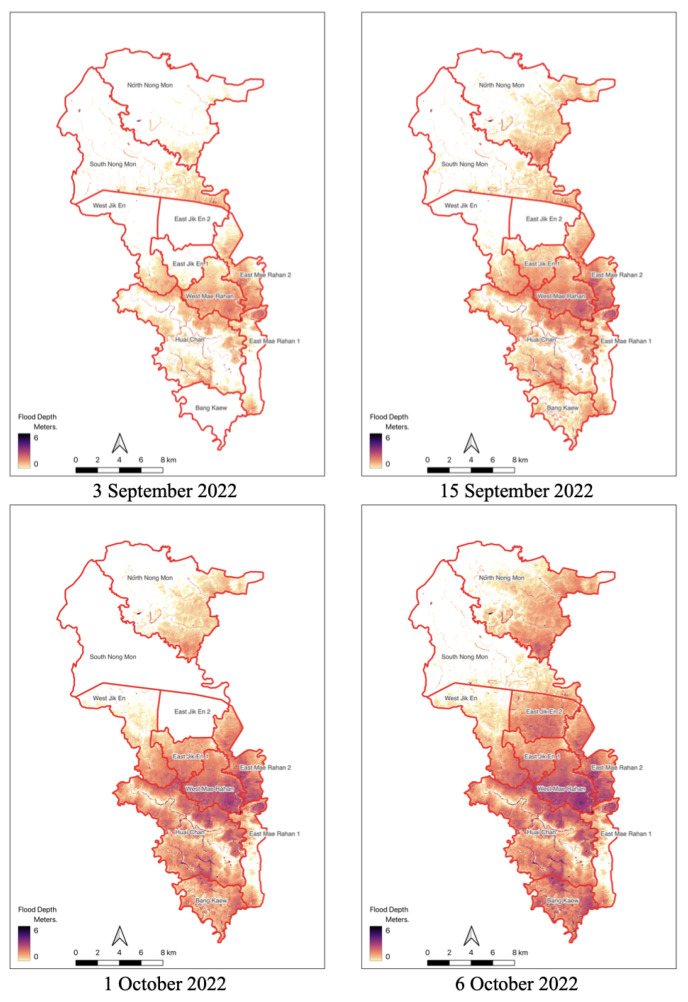
Flood area and flood depth generated from the simulation model in the study area.

This paper estimated water depth levels in near real time and created flood maps using a flood analysis algorithm, as mentioned earlier. Complete data from all 10 stations was only accessible until 6 October 2022, due to damage at Station 7 and equipment collection from Stations 4 and 6 on 7 October 2022. In order to represent the scope and evolution of floods, flood simulations were run on 3 September, 15 September, 1 October, and 6 October 2022. The generated flood depth maps from the simulation are illustrated in Figure 11.

According to the data, flood coverage increased gradually until it peaked in early October, when water depths in certain locations reached 6 m. The most-affected areas of the project region were in the center and south, which included the Bang Kaew, Huai Chan, West Jik En, and the western and eastern Mae Rahan fields.

The size of flood areas grew dramatically throughout the course of the investigation. According to the simulation, South Nong Mon was unaffected by the flooding, while the Huai Chan field had a massive area by 6 October 2022.

### 3.2. Flood from the Model Compares to Flood from Satellite Images

**Figure 12:**
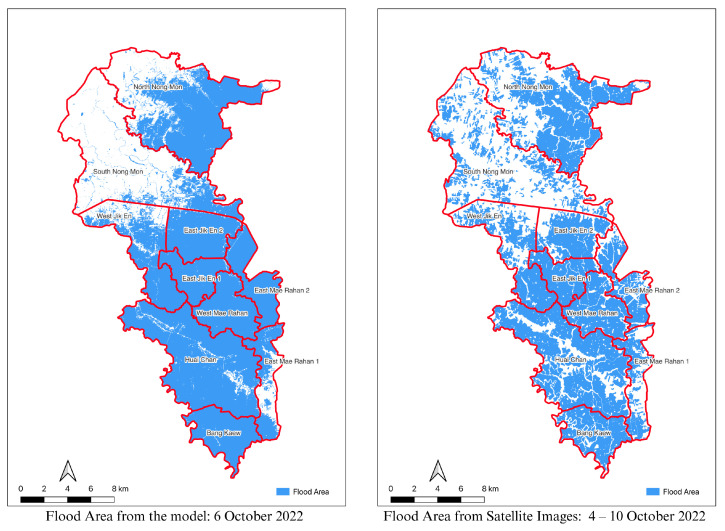
The flood area from the model compares to the flood map derived from satellite images.

The comparison of flood maps produced by satellite imagery analysis and simulations reveals both parallels and discrepancies in the forecast of flood extent. We produced a simulated flood map for 6 October 2022, for this study and contrasted it with flood maps created from satellite imagery taken between 4 October and 10 October 2022. This time range was required because a single satellite overpass could not fully cover the study area due to revisit limitations. The satellite-derived flood maps used for comparison were obtained from secondary data provided by the Geo-Informatics and Space Technology Development Agency (GISTDA). These maps were generated through the interpretation of multi-source satellite imagery, including Sentinel-1, Sentinel-2, RADARSAT, COSMO-SkyMed, and other SAR (Synthetic Aperture Radar) datasets. The data is publicly available as open data and is released by GISTDA during major flood events for public use and decision support. The overall flood boundary trends from both sources were aligned, as seen in Figure 12.

Table 4 compares flooded areas from the flood model (6 October 2022) and satellite imagery (4–10 October 2022). The model estimated a total flooded area of 238.83 sq km, while the satellite image showed 213.22 sq km. Most locations show similar patterns, though some discrepancies exist—such as Bang Kaew, where the model predicted a larger area, and South Nong Mon, which showed flooding only in the satellite data. These differences may result from observation timing or satellite limitations.

In comparison to satellite data observations as shown in Figure 11, the flood simulation model anticipated much bigger flooded areas in specific places. In some cases, the model predicted floods when none existed, while in others, it failed to detect flooding that was captured on satellite images. This reveals disparities between simulated and actual flood occurrences, indicating possible areas for improvement in model accuracy and calibration.

These disparities highlight potential deficiencies in simulation models or variations in the techniques used to collect data for satellite and ground-based observations. This simulation model solely employed ground-level data from sensor locations, which may not have represented the catchment area’s lowest points. This technique might ignore key topographic variables, resulting in mistakes in estimating water flow and flood extent during real-world flood occurrences. Satellite observations, on the other hand, give near real-time data but are susceptible to atmospheric conditions, sensor limits, and picture resolution constraints, which can result in mistakes in outlining flood borders. Furthermore, delays in satellite overpasses or cloud cover during data collection might result in incomplete or inaccurate flood-area estimations.

## 4. Discussion

The developed system was evaluated in terms of both its sensor performance and its ability to generate reliable flood models. This section discusses the advantages and limitations of the system in two main aspects. Section 4.1 focuses on the low-cost water-level sensor, highlighting its technical capabilities, economic feasibility, and field deployment results. Section 4.2 presents the flood model, emphasizing the accuracy of simulated flood maps, their comparison with satellite-derived data, and the challenges arising from terrain complexity and sensor placement.

### 4.1. Low-Cost Water-Level Sensor

The ultrasonic sensor has a measurement range of approximately 400 cm. Although this range is not exceptionally high, it is well suited for areas with moderate flooding, such as the low-lying Bang Rakam model area. While expensive sensors can be installed at greater heights, their high costs make widespread deployment impractical. For instance, in this project, installing traditional sensors priced at 150,000 Thai Baht each across 10 stations would require a budget of 1,500,000 Thai Baht, which may not justify the investment in high-cost equipment for this area. Moreover, the water management operations for the Bang Rakam model area are conducted over a period of only 3–4 months per year. Investing in high-cost sensors for such a limited duration may not be economically viable. In contrast, the use of low-cost sensors developed in this study (each station costs approximately 35,000 Thai Baht) facilitates broader and more cost-effective deployment, enhancing the coverage and effectiveness of flood monitoring in the region. The developed water-level sensor utilizes the Waterproof Ultrasonic Module (JSN-SR04T), which operates on low power consumption, making it suitable for battery-powered applications. Field tests conducted in flood-prone areas, along with controlled experiments using a water-level simulation in a glass tank, demonstrated that the sensor provides accurate water-level measurements. When compared with manual readings from a staff gauge, the sensor exhibited reliable performance. Given its high accuracy and low cost, this sensor proves to be a cost-effective solution for future water-level monitoring applications.

The developed water-level sensor operates on low power, making it ideal for battery-powered applications. It provides ±1 cm accuracy with millimeter-level resolution, ensuring precise water-level readings. These irregularities may be attributed to various factors, including sensor errors at certain times and the detection of objects other than the water surface, such as vegetation. A 12-month field test of the sensor enclosure showed that in the early stages, before flooding occurred, the sensor occasionally misread distances due to interference from grass or weeds. However, once the study area was inundated, the system exhibited high stability, accuracy, and durability, with only minor errors. These minor discrepancies are expected in highly sensitive distance sensors, which may register false readings when objects pass through or obscure the actual water level.

The implementation of water-level sensors enables continuous data collection every hour. This allows users to review historical records and visualize trends through graph-based analysis. It significantly improves efficiency compared to traditional manual staff gauge readings, which require personnel to conduct daily measurements—typically providing a maximum of two readings per day. A 12-month field deployment, covering both pre-flood and actual flood periods, demonstrated that the sensors operated effectively under real-world conditions, exhibiting durability and long-term reliability. However, occasional failures in data transmission to the central server were observed. These issues were primarily caused by two factors. The first was power supply limitations: Since the sensors rely on solar energy, cloudy or rainy days sometimes prevent them from storing sufficient energy, leading to temporary sensor inactivity. A recommended solution is to increase the battery capacity to support multiple days of operation without sunlight.

The second is internet connectivity issues: As the IoT-based sensors depend on 4G or 5G networks for data transmission, network disruptions occasionally prevent data from reaching the central server. This problem is further exacerbated when combined with power shortages. A potential solution involves conducting a network signal assessment at installation sites to determine if switching network providers is necessary. Despite these challenges, the developed system has proven to be an effective and scalable solution for flood monitoring. It significantly improves data availability while reducing the need for manual labor. Further enhancements to power and connectivity are expected to improve its reliability and operational efficiency. These improvements will also strengthen the system’s credibility and dependability during critical disaster scenarios, where timely and accurate data is essential for decision-making and public safety.

### 4.2. Flood Model

This research employs an automated program that retrieves real-time water-level data from a database to generate hourly flood maps, aligning with the sensor’s data collection intervals. The resulting maps visualize flood extent and water depth, which are then displayed on web and mobile applications for monitoring and decision-making. Additionally, all generated maps are stored on a central server, ensuring convenient access for future reference and verification. To evaluate the accuracy of the flood model developed in this study, satellite-derived flood extent data from GISTDA was used for comparison. The analysis revealed key differences between the two datasets, which can be attributed to the following factors:

- Temporal limitations of satellite data: Satellite-derived flood data relies on satellite overpasses, which occur approximately every 7 days to ensure full coverage of Thailand. As a result, there are gaps in data availability for certain periods, leading to missing or outdated information. Furthermore, the mosaicking process, which combines images from different times, may not reflect real-time flood conditions, especially if water levels have changed due to drainage or natural fluctuations within the 7-day interval [13,14].
- Atmospheric errors in satellite image processing: Satellite-based flood mapping is dependent on image processing algorithms that apply predefined classification rules. However, atmospheric variations (e.g., cloud cover, haze, and lighting conditions) introduce errors in flood extent detection as images captured on different days may yield inconsistent results in flood-area estimation.
- Exclusion of permanent water bodies: The publicly available satellite-derived flood maps typically exclude permanent water bodies such as rivers, canals, and reservoirs as these are not considered part of the temporary flood extent. When comparing these datasets with the flood model developed in this study, this exclusion contributes to the observed differences in flood extent.
- Lack of water depth information in satellite data: One major limitation of satellite-derived flood maps is the inability to provide water depth measurements. Unlike the sensor-based approach in this research, which generates flood depth maps, satellite data only indicates the presence or absence of floodwaters. The flood depth information obtained from sensors enables the calculation of floodwater volume, which is crucial for effective water management and disaster response.

Although the system shows promising results, its accuracy is limited by complex terrain and challenges in sensor deployment. Discrepancies between model outputs and satellite data highlight issues with topography, sensor coverage, and installation constraints. The following limitations were identified and are discussed below.

- Limitations of sensor placement in complex topography: A significant difference between the modeled flood area and the satellite image was observed in the “South Nong Mon” area. This discrepancy is primarily attributed to the sub-catchment’s intricate topography, which exhibits substantial elevation differences, with the northwestern section being higher and sloping towards the lower southeastern area. In such varied terrain, a single representative water-level sensor may not accurately capture the nuanced flooding conditions across the entire sub-catchment. The spatial variability in elevation influences localized water accumulation and flow patterns, meaning the sensor’s readings, while valid for its immediate vicinity, might not fully reflect the broader flood extent as observed by satellite.
- Sensor placement was limited by land ownership and access constraints, preventing installation at the lowest elevation points in some catchments. This may reduce measurement accuracy in certain areas. Additionally, although sensors were mounted 3.5 m above ground, surrounding terrain at lower elevations led to simulated water depths exceeding sensor height. This reflects real topographic variation but remains a limitation for interpreting localized flood extent.
- This study is limited by the relatively small number of sensors (only ten across the entire study area) and their spatial distribution. Due to practical constraints such as land ownership and accessibility, sensor placement was not always optimal. Some flood-prone micro-catchments may lack adequate monitoring coverage, which can reduce the precision of flood simulations, especially in areas with rapid hydrological changes. A denser and more evenly distributed sensor network would likely enhance model calibration and improve flood prediction accuracy in future implementations.

## 5. Conclusions

The study developed and evaluated an IoT-based flood monitoring system integrated with GIS for lowland agricultural areas. The system addresses key limitations of satellite-based approaches, particularly delays and cloud cover interference, by providing near real-time, accurate, and reliable water level and flood extent data. The results demonstrate that the system achieves comparable accuracy to high-cost commercial sensors while offering a significantly more cost-effective solution.

The low-cost ultrasonic water-level sensors, combined with automated flood simulation models, proved effective in generating flood depth maps and enhancing monitoring capabilities in the Bang Rakam model area. Despite challenges such as power supply interruptions, network connectivity issues, and limited sensor deployment in complex terrain, the system consistently delivered reliable results in field tests.

Overall, the developed system provides a scalable and affordable solution for real-time flood monitoring and decision-making. With further improvements in sensor distribution, power management, and network reliability, the system holds strong potential to support disaster preparedness and water management strategies in flood-prone agricultural regions beyond the case study area.

## Figures and Tables

**Table 1:** Station information, including coordinates and ground height.

Station No.	Field Name	Latitude	Longitude	Ground Height (m MSL)
1	Bang Kaew	16°47′01.22″ N	100°06′14.74″ E	40.695
2	East Jik En 1	16°56′38.60″ N	100°04′35.30″ E	40.288
3	East Mae Rahan 1	16°53′31.60″ N	100°07′53.10″ E	40.642
4	West Mae Rahan	16°52′13.62″ N	100°08′10.68″ E	40.663
5	North Nong Mon	16°59′57.10″ N	100°05′44.82″ E	43.208
6	Huai Chan	16°50′59.83″ N	100°06′08.45″ E	40.073
7	West Jik En	16°53′40.23″ N	100°03′10.34″ E	41.023
8	East Mae Rahan 2	16°52′09.00″ N	100°07′50.03″ E	40.167
9	East Jik En 2	16°54′11.79″ N	100°04′39.28″ E	42.123
10	South Nong Mon	16°58′34.99″ N	100°05′47.52″ E	42.601

**Table 2:** Structure of the sensor data table used in the database.

Name	Description	Data Type	Length	Key	Reference
id	Record ID	integer	10	PK	-
id_station	Station ID	integer	10	-	-
station_name	Station Name	integer	10	-	-
distance	Distance to water surface	double	-	-	-
sensor_date	Data collection date	date	-	-	-
sensor_time	Data collection time	time	-	-	-

**Table 3:** Monthly schedule of activities.

Period Time	Activity
January	Installed and Test
February	Installed and Test
March	Installed and Test
April	Installed and Test
May	Installed and Test
June	Installed and Test
July	Installed and Test
August	Flooded data collection
September	Flooded data collection
October	Flooded data collection
November	Flooded data collection
December	Installed and Test
January	Installed and Test

**Table 4:** Comparison of flooded areas from model and satellite image (October 2022).

Station No.	Field Name	Flooded Area (Square km) from Flood Model on 6 October 2022	Flooded Area (Square km) from Satellite Image on 4–10 October 2022
1	Bang Kaew	49.00	15.79
2	East Jik En 1	19.62	13.34
3	East Mae Rahan 1	23.69	8.28
4	West Mae Rahan	20.26	16.96
5	North Nong Mon	21.55	44.33
6	Huai Chan	60.81	47.23
7	West Jik En	11.01	19.36
8	East Mae Rahan 2	18.11	11.65
9	East Jik En 2	14.78	14.97
10	South Nong Mon	0.00	21.32
Total	–	238.83	213.22

Flooded areas are measured in square kilometers (sq km).

## Data Availability

The data and code are available on GitHub (main branch, commit 6064ae5, Python 3.9.7) (https://github.com/meoneogeo/bangrakammodel, accessed on 10 June 2025).

## Abbreviations

The following abbreviations are used in this manuscript:

GIS	Geographic Information System
SAR	Synthetic Aperture Radar
DEM	Digital Elevation Model
DTM	Digital Terrain Model
DSM	Digital Surface Model
RID	The Royal Irrigation Department
GIC	Graphic Information Camera
CCTV	Closed-Circuit Television
AI	Artificial Intelligence
IoT	Internet of Things
MQTT	Message Queuing Telemetry Transport
m.MSL	Meter above Mean Sea Level
